# Fractional Factorial Design Study on the Performance of GAC-Enhanced Electrocoagulation Process Involved in Color Removal from Dye Solutions

**DOI:** 10.3390/ma6072723

**Published:** 2013-07-10

**Authors:** Marius Sebastian Secula, Igor Cretescu, Benoit Cagnon, Liliana Rozemarie Manea, Corneliu Sergiu Stan, Iuliana Gabriela Breaban

**Affiliations:** 1 Faculty of Chemical Engineering and Environmental Protection, Gheorghe Asachi Technical University of Iasi, 73, Prof. Dimitrie Mangeron Street, Iasi 700050, Romania; E-Mails: mariussecula@ch.tuiasi.ro (M.S.S.); icre@ch.tuiasi.ro (I.C.); stancs@tuiasi.ro (C.S.S.); 2 Research Center on Divided Matter, CNRS—University of Orléans, 1B, rue de la Férollerie 45071 Orléans cedex 2, France; E-Mail: benoit.cagnon@cnrs-orleans.fr; 3 Faculty of Textile, Leather and Industrial Management, Gheorghe Asachi Technical University of Iasi, 29, Prof. Dimitrie Mangeron Street, Iasi 700050, Romania; E-Mail: lmanea@tex.tuiasi.ro; 4 Department of Geography, Faculty of Geography and Geology, Alexandru Ioan Cuza University of Iasi, 20A, Blvd. Carol I, Iasi 700505, Romania; E-Mail: iulianab@uaic.ro

**Keywords:** electrocoagulation, granular activated carbon, coupling process, fractional factorial design, Acid Blue 74, Basic Red 1, Reactive Black 5, alternating pulse current

## Abstract

The aim of this study was to determine the effects of main factors and interactions on the color removal performance from dye solutions using the electrocoagulation process enhanced by adsorption on Granular Activated Carbon (GAC). In this study, a mathematical approach was conducted using a two-level fractional factorial design (*FFD*) for a given dye solution. Three textile dyes: Acid Blue 74, Basic Red 1, and Reactive Black 5 were used. Experimental factors used and their respective levels were: current density (2.73 or 27.32 A/m^2^), initial pH of aqueous dye solution (3 or 9), electrocoagulation time (20 or 180 min), GAC dose (0.1 or 0.5 g/L), support electrolyte (2 or 50 mM), initial dye concentration (0.05 or 0.25 g/L) and current type (Direct Current—*DC* or Alternative Pulsed Current—*APC*). GAC-enhanced electrocoagulation performance was analyzed statistically in terms of removal efficiency, electrical energy, and electrode material consumptions, using modeling polynomial equations. The statistical significance of GAC dose level on the performance of GAC enhanced electrocoagulation and the experimental conditions that favor the process operation of electrocoagulation in APC regime were determined. The local optimal experimental conditions were established using a multi-objective desirability function method.

## 1. Introduction

The United Nations Organization and World Water Council forecasted in 2003 [1] a possible world water crisis, and the need for sustainable development goals was admitted. Nowadays, fresh and drinkable water is still used in processes where there is no need of high quality water, so wastewater recycling would be possible after appropriate treatment. This is the main reason why some European countries are modifying legislative norms concerning water recycling management [2]. Wastewater treatment applied at source would lead to the possibility of reusing processed water. Moreover, it could prevent the mixing of refractory compounds with other types of effluents, which means a reduction of treated water volume and, implicitly, of treatment costs.

One of the most important classes of pollutants is represented by dyes. When found in reach water, their synthetic character and complex molecular structure make them more stable and harder biodegradable pollutants [3,4]. In recent years, water recycling in textile industry has become a necessary element [5]. Gupta Suhas [6] pinpointed the need of some systematized studies on separation/degradation processes of dyes, and the consideration of wastewater treatment at the source underlines the importance of developing technologies that are simple, reliable, adaptable and relatively cheap. In this way, an increasing interest has been shown in combining processes such as electrocoagulation, electro-oxidation, adsorption, ozonation [7] and reverse osmosis [8].

The electrocoagulation (*EC*) process is known to be faster and more economical than the classical process of chemical coagulation [9]. However, Avsar *et al*. stated that the main disadvantage of conventional *EC* consists in the formation of an impermeable oxide film on the cathode [10], resulting in higher energy consumptions and lower efficiencies [10,11,12,13,14]. This prevents the effective current transfer between the anode and cathode so that the performance of the *EC* reactor decreases. One of the suggested solutions consists in the polarity changing of electrodes resulting in the so-called “self-cleaning” of electrodes. The use of alternating current in *EC* system delays the passivation of cathode and anode deterioration, phenomena met in direct current systems, and thus, ensures a reasonable lifetime of electrodes [11].

Only recently, Eyvaz *et al*. [15] and Mao *et al*. [16] claimed that the advantage of using alternating current in *EC* systems consists in a diminution in energy consumption and superior treatment efficiency. Nevertheless, Keshimirizadeh *et al*. [17] reported that *EC* operated in Alternative Pulsed Current (*APC*) mode, in comparison to Direct Current (*DC*) mode, for removing Cr(VI) ions results in no enhancement of the process performance either in terms of removal efficiency, or in terms of energy consumption. These studies adopted the familiar “one-at-a-time” approach, in which all factors are held constant while one factor is varied. We consider that a more systemized approach, such as statistical methods of investigation, would elucidate the effect of APC use and its favorable experimental conditions on the *EC* performance.

Another possibility to enhance conventional *EC* systems has been suggested by Narayanan and Ganesan, who reported the use of granular active carbon. They showed that a hybrid *EC*-sorption system might be a more efficient and faster separation technique compared to conventional *EC* [18]. However, this approach is new only in light of coupling GAC adsorption with a soluble-electrode electrochemical technique. The advantages of sorption processes conducted in a field of electric current are well-known either in connection with electrosorption [19] or electrooxidation [20] phenomena usually met in applications, such as the regeneration of spent GAC.

Textile effluents present complex compositions, which is why the use of a synthetic aqueous solution facilitates the assessment of a treatment process for emerging technology. The pollutant model used in this *FFD* study, Acid Blue 74, was selected as the most toxic and persistent of the three dyes considered. Acid Blue 74 is an indigoid dye, which is widely employed in textile industry in the dyeing of polyamide and protein fibers [21]. Though, toxicity and environmental data for the considered dyes are limited, it is well-known that the presence of these organic aromatic compounds in the aquatic media could lead to long-term adverse effects [22,23,24].

The aim of this study is to provide a better understanding of the phenomena occurring in a GAC-enhanced *EC* system applied for dye removal from aqueous solutions, as well as to statistically assess the effect of current types. This work presents a systematic and extensive examination of the effects of seven parameters on the performance of *EC* process enhanced by GAC adsorption.

## 2. Experimental

### 2.1. Materials

The GAC material used in this study wasL27 (Pica Jacobi, France). Before use, the adsorbent was washed several times with water and then dried at 120°C for 24h. Nitrogen adsorption-desorption isotherms obtained at 77 K allowed to determine the following textural characteristics: specific microporous volume of 0.57 cm^3^/g, mean pore size of 1.85 nm, specific external surface of 444 m^2^/g, specific microporous surface of 616 m^2^/g and specific total surface of 1060 m^2^/g. In terms of chemical surface, L27 has acidic surface with a pH_PZC_ value of 3.0. This GAC has a high specific microporous volume and external surface, which could favor intraparticle diffusion as we have reported in our previous work [25]. This activated carbon was especially used to liquid adsorption and it was interesting to use it in a coupling EC/AC. In terms of granulometry, the L27 has a mean particle size of 718 µm, which is easier to separate through a filtration stage, in comparison with a powdered activated carbon.

Dyes listed in Table 1 were chosen due to their various characteristics. They were used as received. Acid Blue 74 (55% dye content) and Reactive Black 5 (55% dye content) were purchased from Sigma Aldrich; and Basic Red 1(99% dye content) was purchased from Dalian Chemicals Co., Ltd. (Dalian, China).Thus, *EC* separation features were tested for both acid and basic dyes. Also, it is emphasized the ease of *EC* technology to remove azo dyes. Dye Solutions of 1L volume were prepared before each experimental run by dissolving precisely-weighted amounts of commercial dye in ultra-purified water (resistivity of 18.2 Mohm·cm at 25 °C). NaCl (A.R. Lach-Ner, Neratovice, Czech Republic) was used as background electrolyte.

**Table 1:** Structural chemical formula of investigated dyes: Acid Blue 74; Basic Red 1 and Reactive Black 5.

Color index	Structural formula	λ_max_, nm	M, g/mol
Acid Blue 74	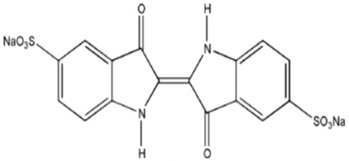	612	466.34
Basic Red 1	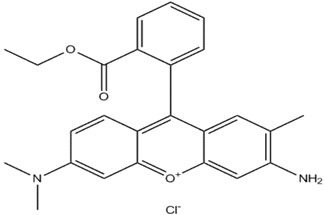	535	436.94
Reactive Black 5	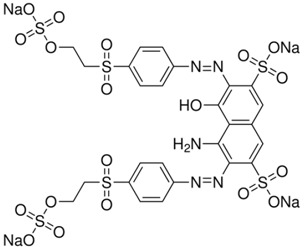	598	991.82

### 2.2. Electrocoagulation Experiments

**Figure 1:**
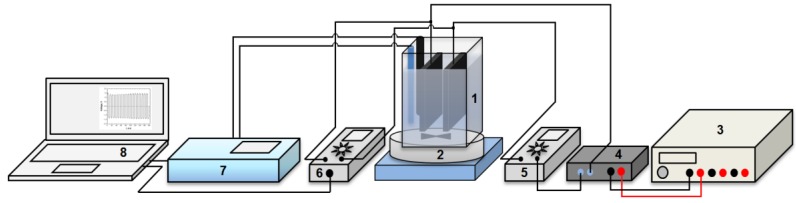
Experimental set-up operated in Alternative Pulsed Current *(APC)* mode. [1—electrocoagulation (EC) cell; 2—magnetic stirrer; 3—Direct Current (DC) power supply; 4—polarity changer; 5—ammeter; 6—data logging voltmeter; 7—multi-parameter analyzer; 8—PC-computer].

Experimental tests were conducted in an *EC* cell provided with two facing plan plate electrodes. For each electrode, the active surface was of 183 cm^2^. In our preliminary studies it was observed the superior performance in dye removal of mild steal-based electrode configurations in comparison to aluminum-based configurations. Therefore, in the present study two plan plates of mild steel were employed. When the *EC* reactor was operated in *DC* mode, the electrodes were connected directly to a digital *DC* power supply (IT6322, 0–30 V; 0–3 A; ITECH, Nanjing, China). For this type of current, the experimental set-up is similar to that described in our previous work [24]. In case of *APC* mode, an automatic polarity changer was employed in the electrical circuit as described in Figure 1.

**Figure 2:**
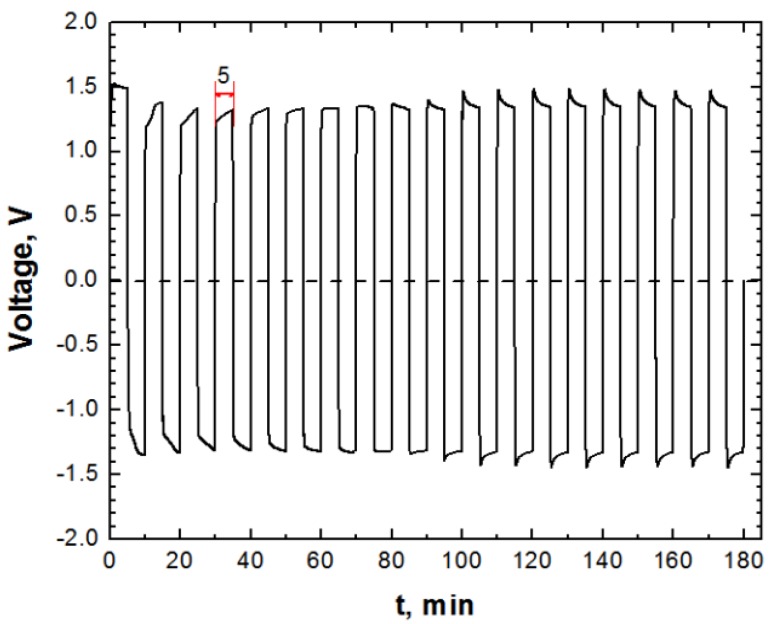
Evolution of voltage during *EC* conducted under alternating rectangular pulse current (*i* =15.025 A/m^2^, pH =6, GAC dose =0.3 g/L, *C*_NaCl_ =26 mM, *C_i_* =150 mg/L).

The experiments were carried out in batch mode according to the procedure described in detail in [24]. A VC530 Voltcraft data-logger multimeter connected to a computer was used to measure with one reading per second the cell voltage. Figure 2 shows how the voltage of *EC* cell varies when operated in *APC* mode. Solution pH and conductivity were measured by means of a *PC*-connected C863 Consort multi-parameter analyzer.

In the coupling process experiments, GAC was introduced in the range 0.1–0.5 g/L under mechanical stirring into 1L of the dye solution directly in the electrocoagulation cell (Figure 1). More details are available in our previous work [25]. After the experiment, the samples were filtered through a 0.45 mm membrane filter, and then analyzed.

The electrodes were polished with emery paper of various grades, washed with dilute H_2_SO_4_ and then with distilled water before each experimental run. After drying, electrodes were weighed before and after *EC* by means of an Acculab ATL-224-I analytical digital balance (accuracy of 0.1 mg) to estimate the amount of electrode dissolved. All experimental runs were performed at room temperature of about 25°C.

The color removal efficiency (*Y*, %) was calculated from:

(1) $$Y=\frac{C_{i}-C}{C_{i}}\cdot 100$$

where *C_i_* is the concentration of dye before treatment (mg/L); and *C* is the concentration of dye after *t* minutes of treatment (mg/L).

The concentrations of each dye were determined using the initial calibration curves which were recorded after spectrophotometric measurements of the solution absorbance for each dye standard concentration at the specific wavelength corresponding to the maximum absorption of each dye (see λ_max_ [nm] in Table 1).

### 2.3. Energy and Electrode Material Consumptions and Costs

The most important costs of electrocoagulation technology are related to the consumption of electrical energy and electrode material.

When *EC* tests are conducted in galvanostatic regime, *i.e.*, the current intensity is maintained constant, the cell voltage varies. Therefore, the energy consumption related to the amount of removed dye, unit energy demand (*UED*, kWh/kg) [26,27], can be determined by means of the following relationship:

(2) $$UED=\frac{I\cdot \int_{0}^{t}U\cdot dt}{1000\cdot V\cdot C_{i}\cdot \frac{Y_{t}}{100}}$$

where *U* is the cell voltage, (V); *I*—current intensity, (A); *t*—time, (h); *V*—volume of treated solution, (m^3^); *Y_t_*—color removal efficiency at time *t*, (%).

The generation of coagulant during *EC* process leads to the consumption of electrode material that can be estimated based on Faraday’s law [28]:

(3) $$UEMD=\frac{I\cdot t\cdot A}{n\cdot F\cdot V\cdot C_{i}\cdot \frac{Y_{t}}{100}}$$

where *UEMD* is the unit electrode material demand, (kg/kg); *t*—time, (s); *n*—number of electrons involved in oxidation/reduction reaction; *F*—Faraday’s constant, (*C/*mol); *A*—atomic mass of electrode material, (g/mol).

Electrical operational costs (*EOC*s) of the electrocoagulation dye wastewater can be calculated by means of Equation (4) on the basis of the amount of energy consumption and consumed materials [27].

(4) EOC=EEC+EMC=UED⋅EEP+UEMD⋅EMP

where *EOC* is the electrical operating cost, ($/kg) of dye removed; *EEC*—electrical energy consumption, ($/kg) of dye; *EEP*—electrical energy price, ($/kWh); *EMC*—electrode material cost, ($/kg); *EMP*—electrode material price, ($/g).

### 2.4. Fractional Factorial Design

In order to evaluate the statistical significance of the effects of seven different factors and their interactions on the performance of GAC-enhanced *EC*, a 2^7-3^ fractional factorial design (*FFD*) was developed. A two-level full factorial design for seven factors requires 128 experimental runs. Thus, the advantage of a *FFD* consists in the important reduction in the number of experiments to only 16. The confounded (aliased) factors and interactions for this 2^7-3^ *FFD* are explained in detail in literature [29]. Since three (or more) factor interactions are not likely to be important, the main effects are not confounded with two-factor interactions [30]. High (+1) and low (−1) levels considered for each continuous factor were established based on our prior work [24], as shown in Table 2.

**Table 2:** Predictor variables and their coded and actual values used in the experimental design.

Level	*i* (A/m^2^)	pH	*t* (min)	GAC dose (g/L)	*C*_NaCl_,(mM)	*C_i_*(mg/L)	Current type
−1	2.73	3	20	0.1	2	50	DC
0	15.025	6	100	0.3	26	150	–
+1	27.32	9	180	0.5	50	250	APC

Note:−1 is the low level; 0—center level; +1—high level; *i*—current density; *t*—operating time; *C*_NaCl_—concentration of NaCl; *C_i_—*initial concentration of dye.

Another important goal of this study was to compare the effect of current type on the performance GAC-enhanced *EC* process.

Also, three center points were added for each level of the categorical factor in order to estimate the experimental error and verify whether there is any curvature in the model to be fitted [31]. Experimental data were analyzed statistically by means of Minitab software. Table 3 presents the experimental matrix of the *FFD*.

**Table 3:** Experimental design matrix of the 2^7-3^ fractional factorial design (*FFD*).

Run No.	*A*	*B*	*C*	*D*	*E = ABC*	*F = BCD*	*G = ACD*
	*i*, A/m^2^	pH	*t*, min	GAC dose, g/L	[NaCl] mM	Ci, mg/L	Current type
1	−	−	−	−	−	−	*DC*
2	+	−	−	−	+	−	*APC*
3	−	+	−	−	+	+	*DC*
4	+	+	−	−	−	+	*APC*
5	−	−	+	−	+	+	*APC*
6	+	−	+	−	−	+	*DC*
7	−	+	+	−	−	−	*APC*
8	+	+	+	−	+	−	*DC*
9	−	−	−	+	-	+	*APC*
10	+	−	−	+	+	+	*DC*
11	−	+	−	+	+	−	*APC*
12	+	+	−	+	−	−	*DC*
13	−	−	+	+	+	−	*DC*
14	+	−	+	+	−	−	*APC*
15	−	+	+	+	−	+	*DC*
16	+	+	+	+	+	+	*APC*
17	0	0	0	0	0	0	*DC*
18	0	0	0	0	0	0	*APC*
19	0	0	0	0	0	0	*DC*
20	0	0	0	0	0	0	*APC*
21	0	0	0	0	0	0	*DC*
22	0	0	0	0	0	0	*APC*

Note: “–” represents the low level; 0—center lever; +—high level.

## 3. Results and Discussion

**Figure 3:**
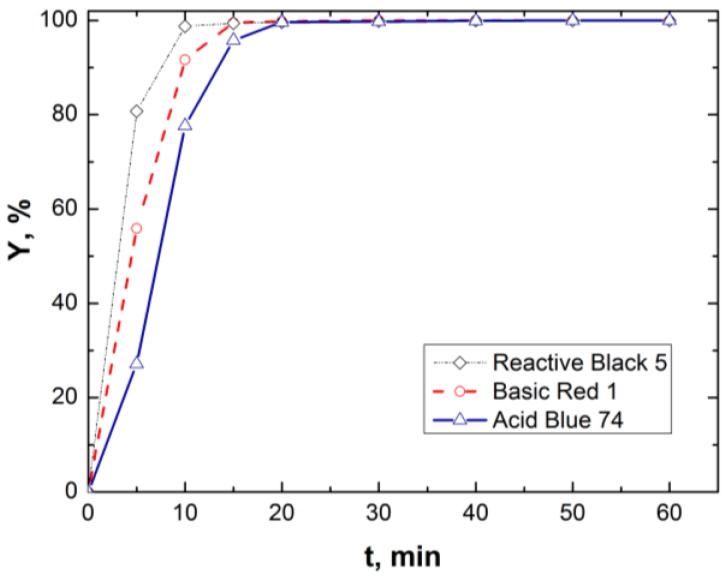
Removal of different dyes by *EC* (*C_i_* = 100 mg/L, *i* = 54.61 A/m^2^, pH*_i_* = 5.5, *C*_NaCl_= 26 mM, *DC* mode).

Figure 3 shows the *EC* performance in terms of color removal efficiency towards three different dyes having various chemical characteristics. For each dye, three experiments were carried out under the same conditions specified in the figure caption. The average values for each set of tests are plotted.

It can be noticed that the behavior of the three dyes considered is not much different, either EC is used to remove an acid dye, such as Acid Blue 74, or a basic dye such as Basic Red1. Moreover, the aqueous solutions containing the most refractory dye considered in this study, Reactive Black 5 decolorized the fastest. Due to its higher persistence compared to the other studied dyes, Acid Blue 74 was chosen as the pollutant model in the present *FFD* study.

Generally, the conclusions drawn from factorial designs depend mainly on the arbitrary-selected ranges of the investigated independent variables. Therefore, it is necessary that the considered levels for each factor (presented in Table 2) be large enough in order to achieve changes that exceed experimental errors. Among the seven considered factors, the type of current is the only categorical factor. Thus, the use of *DC* or *APC* of rectangular wave represented the two levels of this factor.

Previous experience achieved on dye removal from aqueous solutions by conventional *EC* [24] helped us to establish the correct ranges for the six continuous factors. Taking into account the active electrode surface, inter-electrode distance, and safe-operating limits of power supply, we considered a minimum level of 2.73 A/m^2^ and a maximum of 27.32 A/m^2^ for the current density factor. The *EC* time is an important factor due to its influence on the energy and electrode material consumptions, as well as on color removal efficiency. *EC* time factor was investigated in the range of 20 min up to 180 min.

In reference [24] we showed that NaCl is the best electrolyte support for this system. However, the optimal NaCl concentration should be a compromise between the effect of diminishing the energy consumption and that of possible contamination of the treated effluent with chlorides. A range from 2.0 to 50.0 mM ensured a proper investigation of the effects of background electrolyte factor. The pH parameter influences the *EC* performance especially at low values of current density and at the beginning of the process [24]. Therefore, it is estimated that only a wide range of the initial pH such as from 3 to 9 would significantly influence *EC* performance. A low level of 50 mg/L and a high one of 200 mg/L were considered for the initial concentration of dye in agreement with the wastewater treatment studies reported in literature [32,33].

Among the four GAC materials that we studied previously [25], Pica L27 exhibited the best adsorptive properties toward this dye under the conditions of *EC*. In the kinetic study of GAC-enhanced *EC* [25], we found that 0.1 g/L of L27 is the minimum dose where there is a significant improvement of the constant rate compared to that obtained in the case of conventional *EC*. Doses up to 0.5 g/L of L27 lead to a strong increase in constant rate values. However, doses higher than 0.5 g/L of L27 result in a relatively lower augmentation of constant rate values.

**Figure 4:**
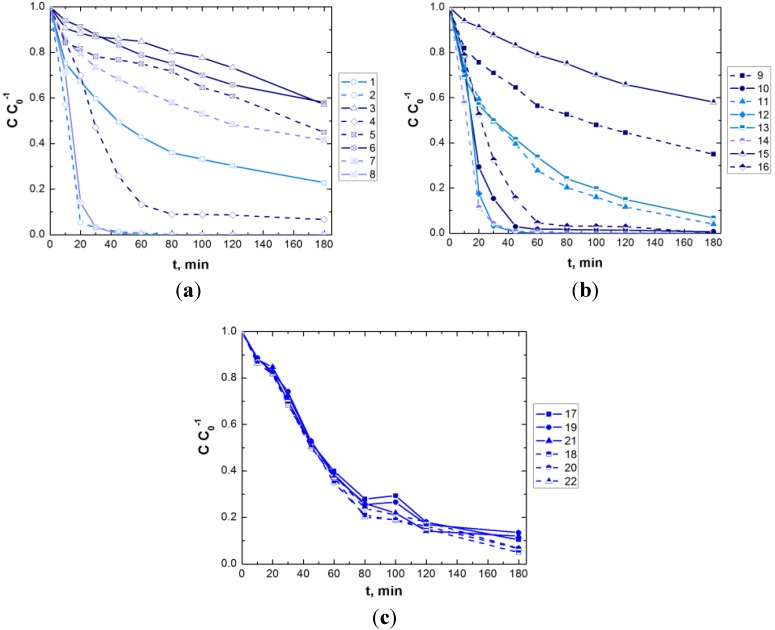
Evolution of color removal efficiency during *EC*/CAG coupling (experimental conditions are depicted in Table 3; solid line—*DC*, dashed line—*APC*; light blue, blue and navy blue lines—low, center and high values of dye concentration).

**Figure 5:**
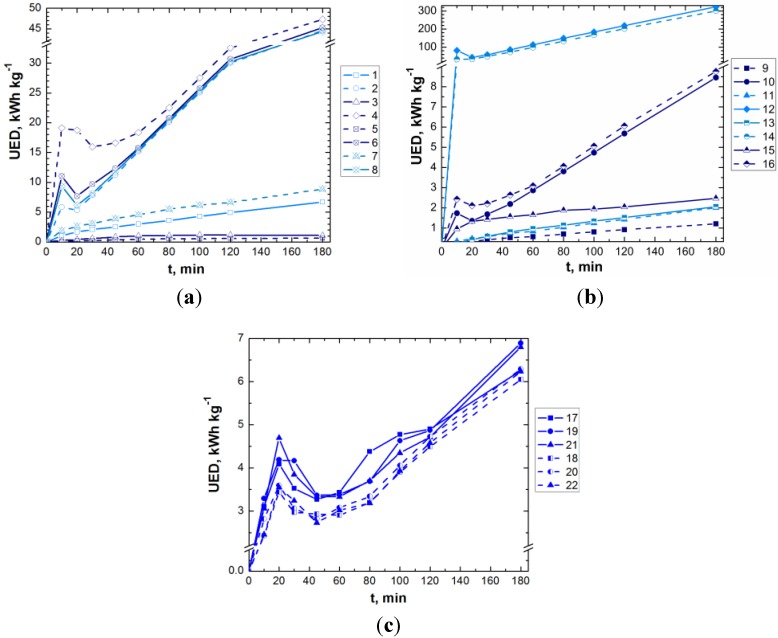
Evolution of unit energy demand (*UED)* during *EC*/CAG coupling (experimental conditions are depicted in Table 3; solid line—*DC*, dashed line—*APC*; light blue, blue and navy blue lines—low, center and high values of dye concentration).

**Figure 6:**
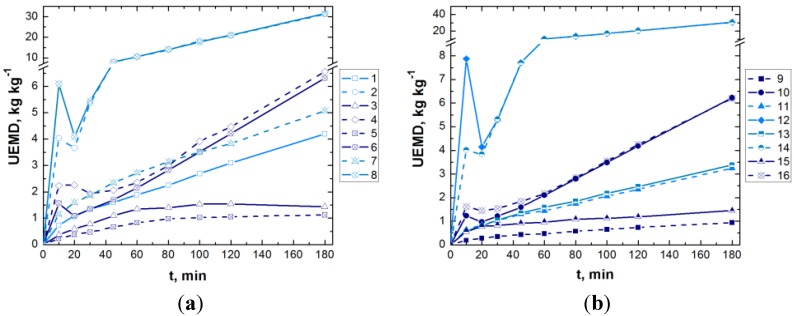

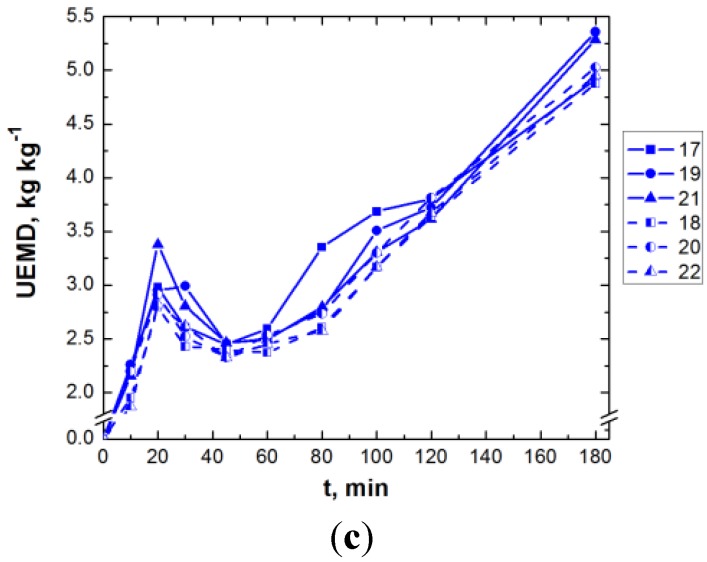
Evolution of unit electrode material demand *(UEMD)* during *EC*/CAG coupling (experimental conditions are depicted in Table 3; solid line—*DC*, dashed line—*APC*; light blue, blue and navy blue lines—low, center and high values of dye concentration).

Figure 4, Figure 5 and Figure 6 present the experimental results obtained in the treatment of aqueous dye solutions by GAC-enhanced *EC* in terms of color removal efficiency, *UED* and *UEMD* as a function of time.

Figure 4c, Figure 5c and Figure 6c show only the results obtained in the center of the experimental matrix (Table 3). As can be noticed, the runs carried out in *APC* mode result in a slightly enhanced decolorization, with lower consumptions of energy and electrode material in comparison with the results obtained for GAC-enhanced *EC* operated in *DC* mode. These observations are also valid for the rest of the runs, the dashed lines (*APC*) being located generally under the solid ones (*DC*). Compared to *DC* mode, the reproducibility of *EC* performance indicators is better in the case of *APC* use.

### 3.1. Effects on the Color Removal Efficiency

Values of color removal efficiency obtained in the randomized 22 runs lie in the range of 11.58% and 100.0%. In order to identify the statistical significant factors and interactions, analysis of variance was employed. It is worth mentioning that main effects represent the difference of the averaged responses for the two levels (+1,−1) of a given factor [34,35]. A two-factor interaction effect can be determined as half the difference between the main effects of one factor at the two levels of the second one [35].

**Figure 7:**
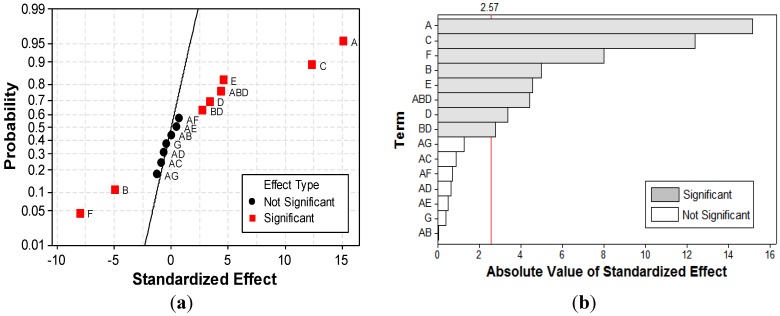
Normal plot of the (**a**) standardized effects; and (**b**) standardized Pareto chart for *Y* response.

Figure 7 depicts the normal plot of the standardized effects and the standardized Pareto chart, *i.e.*, main factors and interactions as a function of the standardized effects. On the Pareto chart (Figure 7b), the standardized effect values of significant (α = 0.95) factors and interactions are higher than the critical value [36]. Though, the Pareto chart allows one to compare the absolute values of the effects of each factor or interaction of the considered *FFD*, normal plot of standardized effects is more accurate in determining, respectively, the significance and insignificance of each effect (Figure 7a). On a normal probability plot of effects, the non-significant ones fall along a straight line, *i.e*., normal distribution, and tend to be centered near zero. In contrast, the following factors caused significant deviations from the straight line [34]. Current density (*A*), contact time (*C*), and initial concentration of dye (*F*) have the most important effects on color removal efficiency. Other significant factors include pH (*B*), concentration of electrolyte support (*E*), GAC dose (*D*), interaction between pH and GAC dose (*BD*), and interaction between current density, GAC dose, and pH (*ABD*).

**Figure 8:**
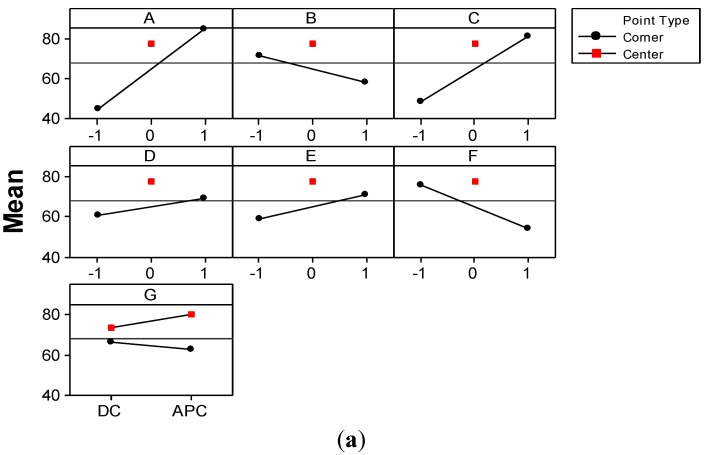

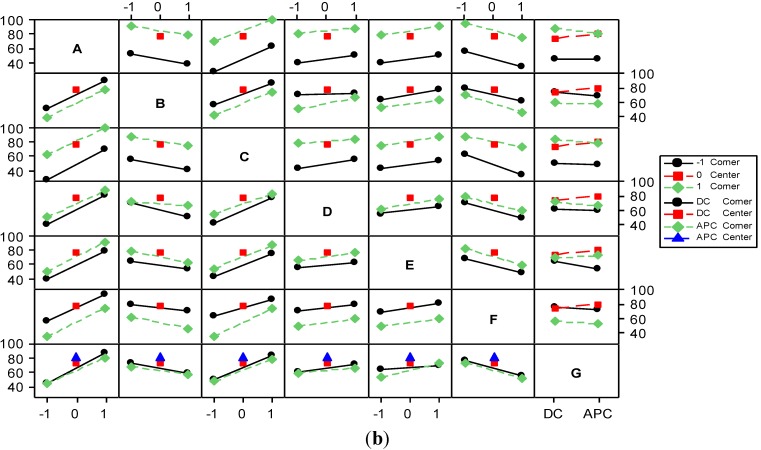
(**a**) Main effects; and (**b**) interactions plots pointing out the effects on color removal efficiency. Solid lines represent low black levels (−1 and *DC*) of the factors; dashed green lines represent high levels (1 and *APC*); dashed red lines represent the center levels (0); left ends of the lines in each plot means low level of underlying factors, and the right ends depicts higher levels.

Only a few authors approached wastewater treatment by conventional *EC* operated in *APC* mode [15,16,17]. Since their results are rather contradictory, one of the goals of the present study was to establish the influence of current type on the performance of GAC-enhanced *EC* technique. To this aim, the main effect plot and interactions plot shown in Figure 8 allow one to analyze in depth the effects of factors considered in the *FFD*.

Table 4 presents a statistical summary of the mathematical models suggested for the considered responses.

**Table 4:** Statistical summary of *Y*, *UED* and *UEMD* models.

Response	Y, %	*UED*, kWh/kg	*UEMD*, kg/kg
Transformation	None	Log	Log
Lack of Fit *p* value	0.336	0.124	0.196
Model *p* value	<0.0001	<0.0001	<0.0001
Model *F* value	101.71	3969.8	1628.9
Curvature *p*-value	<0.0001	0.002	<0.0001
Significant model terms	–	–	–
*	A, B, C, D, E, F, ABD	A, B, C, D, E, F, G, AC, AD, AE, AF, BD	A, B, C, D, E, F, AC, AD, AE, AF, BD
**	BD	AB, ABD	ABD
***	–	–	G, AD
R^2^	0.9854	0.9998	0.9996
R_adj_^2^	0.9693	0.9993	0.8342
R_pred_^2^	0.835	0.9187	0.9982
PRESS^a^	2586	0.9342	0.8183
S^b^	4.788	0.0199	0.0204

*terms significant for a confidence level of 99%; ** terms significant for a confidence level of only 95%; ** terms significant for a confidence level of only 90%; ^a^predicted residual sums of squares; ^b^ square root of the mean square error.

The mean value of color removal efficiency, *Y*, obtained in corner points of *FFD* (Figure 8a) tends to decrease in the case of *APC* use. In contrast, the mean value of color removal efficiency corresponding to the runs performed in the center point increases in the case of *APC* use. The interactions plots (shown in Figure 8b) and ANOVA test (Table 4) shed some light on this issue. For instance, according to the ANOVA test, the interaction between GAC dose and pH parameter (*BD*) is statistically significant for a 95.0% confidence level. GAC dose is more significant in the case of a high value of pH, which is due to the residual acidity of Pica L27 [25]; taking into account that most textile effluents are of an alkaline character [4,5] and that effluents treated by *EC* result in higher values of pH. The benefits of adding a certain amount of this kind of GAC consist in a decrease of pH value, especially at low values of current density, and, therefore, a faster removal of dyes.

According to *FFD* generation structure, this interaction is confounded with *CF* and *EG* interactions [29]. The interaction between the current type (*G*) and the concentration of electrolyte support (*E*) reveals that GAC-enhanced *EC* operated in *DC* mode provides higher values for *Y* response at the low level of *E*. This might be explained by the fact that in *DC* mode the flow of the ions is not perturbed. In contrast, in *APC* mode, the change of electrode polarity leads to a change in the direction of electrophoretic transport of charged particles. Statistically averaged, the current type factor seems to have no influence on the color removal efficiency (Figure 7). However, from Figure 4c and Figure 8a it is clear that the use of *APC* mode has positive effects on *Y* response. In the center of the experimental region, the treated effluent has an adequate conductivity that favors the electrophoretic transport. The middle value of current density range also enables emphasizing the positive effect of *APC*. For instance, at the high level of current density factor, the separation process becomes very fast, and the factor of current type becomes marginal.

Compared to the initial state (when all the terms are included), quality-of-fit indicators of the model obtained after removing the insignificant terms (*G*, *AC*, *AE*, *AF* and *AG*), improved remarkably. As more terms are added to the model, the coefficient of multiple determination, R^2^, increases, which is the reason why we obtained a lower value for this coefficient (0.9911 *vs*. 0.9854) after removing the insignificant terms [36]. In contrast, when significant terms are added or insignificant ones are removed, this slightly improved (0.9627 *vs*. 0.9693) the adjusted determination coefficient, R_adj_^2^, The final suggested model for *Y* response also provides a lower value for the square root of the mean square error (5.727 *vs*. 4.788), a much lower value for PRESS (102087 *vs*. 2586), a good value for R_pred_^2^ (0 *vs*. 0.835) and a high *p* value of the lack of fit (0.336 > 0.05), *i.e*., inadequacy is not significant. Therefore, the model suggested for color removal efficiency correctly describes the experimental data.

The final equation obtained for the decolorization of Acid Blue 74 aqueous solutions by GAC-enhanced *EC* is described by Equation (5) given in Table 5. Though the effects of *AB* and *AD* interactions are not statistically significant, the significant *ABD* interaction implies that these terms should be kept in order to obtain a hierarchical model.

**Figure 9:**
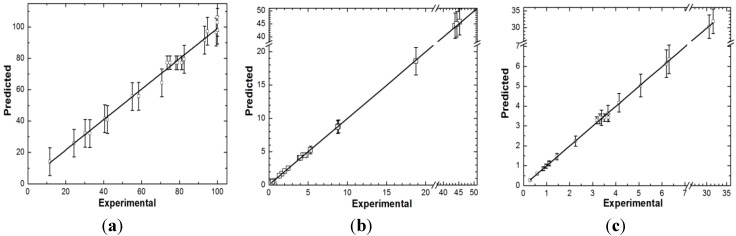
Statistical validation of the models for (**a**) *Y*; (**b**) *UED*; (**c**) *UEMD* responses.

In order to emphasize the statistical validation of the model for color removal efficiency, experimental data were plotted against the predicted ones as presented in Figure 9a.

The experimental values vary within the confidence interval of the values predicted by the suggested model, which supports the fact that the lack of fit is not significant. Therefore, it can be concluded that the model obtained to describe the response of color removal efficiency is adequate. However, Table 5 pinpoints the existence of curvature in the polynomial model. In order to correctly describe the entire experimental region, a second order polynomial model obtained by response surface methodology would be recommended. Only the factors with the highest effects on the response should be considered. However, this task is beyond the goals of the present work.

**Table 5:** Model equations (coded values)*.

Response	Expression	Equation Number
*Y* =	64.71 + 19.96A − 6.55B + 16.31C + 4.45D + 6.01E − 10.55F + 0.04AB − 0.84AD + 3.64BD + 5.81ABD	(5)
Log(*UED*) =	−1.0872 + 0.0575A + 0.0519B + 3.67 × 10^−3^C − 0.119D − 7.53 × 10^−3^E − 2.16 × 10^−3^F − 0.093G + 1.76 × 10^−4^AB + 3.46 × 10^−5^AC + 0.0277AD − 4.11 × 10^−4^AE − 3.47 × 10^−5^AF + 4.82 × 10^−3^AG − 0.06BD − 0.002ABD	(6)
Log(*UEMD*) =	0.597 + 0.341A + 0.058B + 0.0332C − 0.0524D − 0.03E − 0.251F − 0.009G − 0.0051AB + 0.05AC + 0.011AD − 0.021AE − 0.028AF + 0.041AG − 0.051BD − 0.018ABD	(7)

* Note that all continuous factors in fractional factorial design vary from −1 to +1, while the categorical factor G takes only −1 and +1 values.

### 3.2. Effects on the UED Response

Unit Energy Demand (*UED*) response is defined by Equation (2). Values obtained for *UED* response range between 0.03 and 30.04 kWh/kg, and the ratio of maximum to minimum is greater than 10 (898.9), which indicates that a transformation is required [34]. According to the Box-Cox plot for power transformation, Minitab software indicated that the logarithm of response values is the proper transformation.

**Figure 10:**
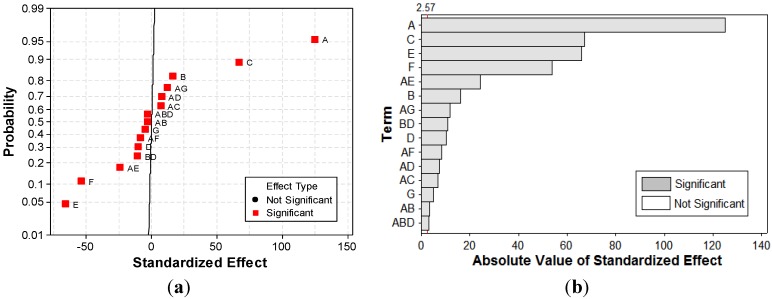
Normal plot of the (**a**) standardized effects; and (**b**) standardized Pareto chart for *UED* response.

Figure 10 presents the normal plot and Pareto chart. It can be noted in the normal plot of *UED* response (Figure 10a) that no straight line crosses the points representing the effects of the coefficients in the model. The Pareto plot in Figure 10b pinpoints that all the main and interaction effects of 2^7-3^ *FFD* have significant effects on *UED* response.

**Figure 11:**
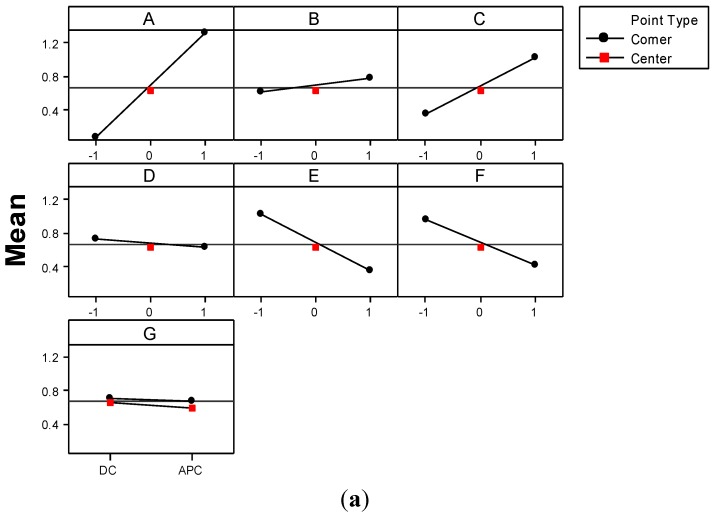

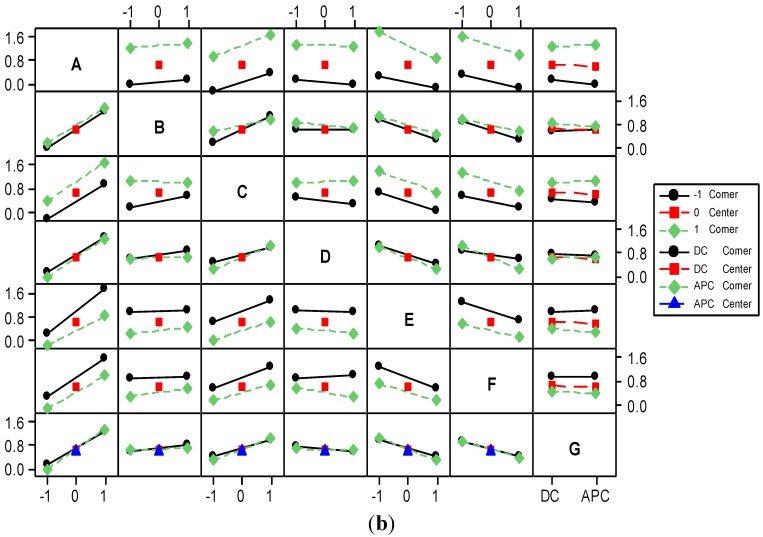
(**a**) Main effects; and (**b**) interaction plots for the effects on *UED* response. Solid lines represent low black levels (−1 and *DC*) of the factors; dashed green lines represent high levels (1 and *APC*); dashed red lines represent the center levels (0); left ends of the lines in each plot means low level of underlying factors, and the right ends depict the higher levels.

Figure 11 presents the main effects and interaction plots for *UED* response.

The current density and contact time factors present the most important effects on the *UED* response. The concentration of background electrolyte factor has a greater effect on *UED* response compared to the initial concentration of dye factor. The current type effect is also significant.

Statistical data support the experimental observations. According to these results, the use of *APC* in *EC* leads to lower energy consumption. As shown in Figure 11a, the mean of *UED* experimental data for *DC* mode is 5.12 kWh/kg. This mean value decreases 10% to 4.65 kWh/kg in the case of *EC* operated in *APC* mode. Therefore, we claim that the use of *APC* in *EC* leads to the diminution of *UED* compared to *DC* mode. In terms of averaged effect, the current type has no significant effect on the response of color removal efficiency. Hence, the advantage of using *APC* in *EC* technology consists of a significant reduction in electrical energy consumption.

The addition of a certain amount of GAC leads to a faster removal of dye. The energy consumption increases proportionally with the GAC dose. This might be due to the adherence between GAC and metal-electrode surface that results in an increase of the total active electrode surface. Even with this slight increase in the energy consumption, the beneficial effect of a faster separation of the pollutant is dominant. Consequently, *UED*, *i.e.*, energy consumption related to the separated amount of dye, decreases with GAC dose. When adding 0.1 g/L of GAC, an experimental mean value of 5.48 kWh/kg was obtained for *UED* response (Figure 10a), while adding 0.5 g/L GAC results in a reduction of 20.8% (4.34 kWh/kg).

Likewise, an acid initial pH favors the removal of dye and leads to a decrease in *UED* response. The mean of the *UED* response values, corresponding to an initial pH of 9, is 5.88 kWh/kg, while in the case of initial pH of 3, the mean is 4.05 kWh/kg. This can be correlated also with the effect of the addition of GAC such as Pica L27 that has an acid character [25]. In case of conventional EC, the magnification in hydroxyl ions generated from water reduction at the cathode, leads to the pH solution increase. When GAC is added into the system, there is a competition between the release of protons due to the acidic functional surface groups of L27 and the generation of hydroxyl ions at the cathode. This is due to water electrolysis and the significantly slower pH increase in the solution.

The adequacy of the model for *UED* is relatively good as emphasized by the statistical summary shown in Table 4 and the experimental *versus* predicted values plot shown in Figure 9b. Equation (6) of the suggested model for *UED* response is given in Table 5.

### 3.3. Effects on the UEMD Response

*UEMD* represents the consumption of electrode material in relation to the mass unity of removed dye as defined by Equation (3). Experimental values obtained for this response range between 0.042 and 47.43 kg/kg (ratio of maximum to minimum is 113.7) requiring also the logarithmic transformation.

**Figure 12:**
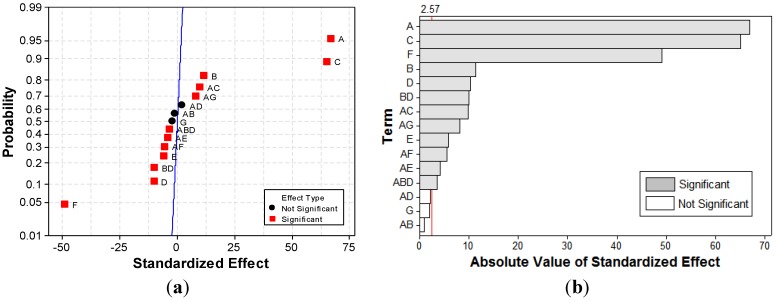
Normal plot of (**a**) the standardized effects; and (**b**) standardized Pareto chart for *UEMD*.

The normal plot of standardized effects (Figure 12a) and standardized Pareto chart (Figure 12b) for *UEMD* response outline that the current type factor and *AB* and *AD* interactions represent the only terms of the model that are not significant. However, taking into account that the superior terms *ABD* and *AG* are significant, insignificant terms were kept in the model to preserve the hierarchic characteristic.

**Figure 13:**
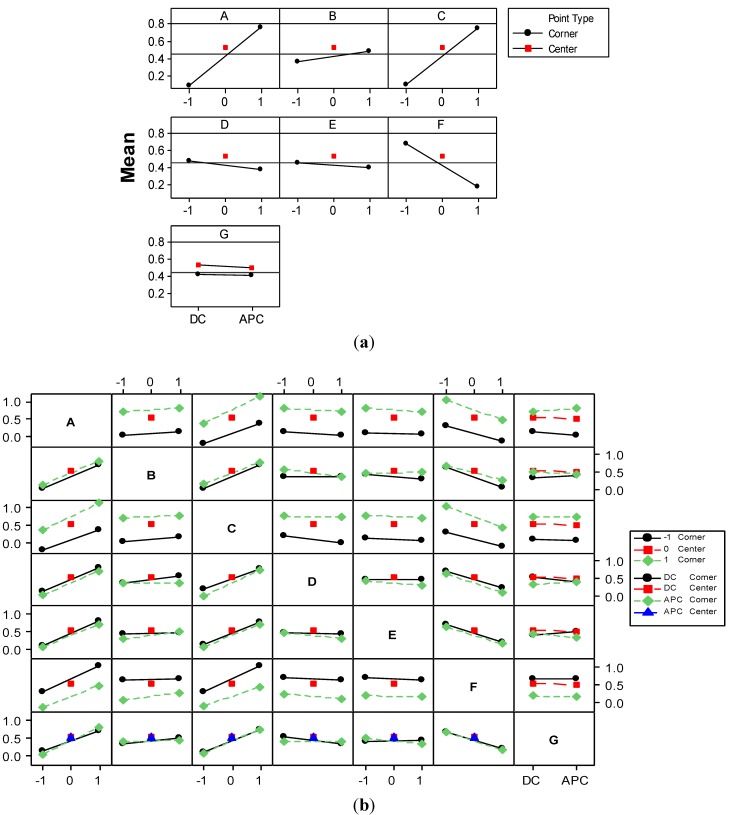
(**a**) Main effects; and (**b**) interaction plots for the effects on *UEMD* response. Solid lines represent low black levels (−1 and *DC*) of the factors; dashed green lines represent high levels (1 and *APC*); dashed red lines represent the center levels (0); left ends of the lines in each plot means low level of underlying factors and the right ends depict the higher levels.

The Pareto chart also emphasizes the strong influence of current density and time factors as well as that of initial dye concentration on the *UEMD* response. This is due to the fact that *UEMD* response is directly dependent on current density and inversely related to electrolysis time and initial concentration of dye. The effects of pH and GAC dose as well as *AG* interaction are significant. This means that electrode material consumption might be diminished if GAC-enhanced *EC* is operated at a certain current density. In order to elucidate the interaction effects, the main effects and interactions plots (Figure 13) obtained for *UEMD* response were analyzed.

As shown in the interaction plot (Figure 13b), while operating *EC* at the low level of current density, the mean of *UEMD* values obtained in *DC* mode is 1.348 kg/kg (Figure 13b), while for APC mode the *UEMD* mean is 1.07 kg/kg. This represents a reduction in the specific consumption of the electrode material of approximately 20%. In contrast, *EC* operated at the high level of current density leads to an increase in *UEMD* mean of 16.4% in case of *APC* mode compared to *DC* mode. Therefore, the beneficial effect of using *APC* mode decreases to about 4% less material consumed on average. This makes the entire main effect of current type (G) to be statistically significant only at a confidence level of 90%.

The interaction of *AG* is confounded with *BF* and *CD* interactions [29]. Figure 13b shows also that a higher initial concentration of dye requires less electrode material dissolved at a low pH value. A higher GAC dose leads to a diminution of *UEMD* at short durations. This is due to the important increase in electrode material consumed at longer durations in relation to the effect of GAC dose.

Table 4 and Figure 9c support that the model suggested for *UEMD* response (Table 5) in Equation (7) is adequate.

### 3.4. Multi-Objective Optimization

Although the suggested models by 2^7-3^ *FFD* are not very reliable to interpolate precisely the entire experimental region, one can use them to estimate the local optimum, which might serve further as a possible center point of the experimental region in a Response Surface Methodology design.

The goals of the optimization of GAC-enhanced *EC* system consist in maximizing the response of color removal efficiency and minimizing *UED* and *UEMD* responses. To solve this kind of multi-objective optimization problem, Derringer and Suich [37] suggested the desirability function (Equation 8) that is one of the most appropriate methods. The overall desirability function, *D*, is the geometric mean of the individual desirability functions [38]:

(8) $$\text{D=}{\left(\prod_{i=1}^{k}d_{i}\right)}^{1/k}$$

with *d_i_* denoting the individual desirability function for each response, and *k* the number of responses, *i.e.*, *k* = 3.

The optimization of the overall desirability function, *D*, implies the maximization of the response of color removal efficiency and the minimization of *UED* and *UEMD* responses. This function is subject to the constraint. In this regard, all the factors take values in the limit of the experimental region explored. Bezerra *et al*. [38] described in detail the methodology of desirability function. Table 6 presents the goals, criteria, optimal values of responses, and values obtained for global and individual desirability functions. Experimental tests were performed to verify the predicted values of responses.

**Table 6:** Optimization criteria and obtained results.

Goals	Criteria	Desirability	Results	
			Predicted	Experimental
–	A,B,C,D,E,F,G∈ Ω	D^a^ = 0.215	–	–
Max(Y)		d_Y_ = 0.432	94.32	92.24 (s.d.^b^ 2.5)
Min(*UED*)		d_log(*UED*)_ = 0.346	0.144	0.178 (s.d.^b^ 0.01)
Min(*UEMD*)		d_log(*UEMD*)_ = 0.242	4.209	4.683 (s.d.^b^ 0.11)

^a^General desirability; ^b^standard deviation of experimental data.

The algorithm of multi-objective optimization Minitab software allowed us to obtain the predicted optimal values. Three confirmation runs were carried out in order to check experimentally the optimal point. Optimal values of responses correspond to a current density of 2.73 A/m^2^, pH value of 3, GAC dose of 0.5 g/L, salt concentration of 50 mM, dye initial concentration of 50 mg/L, duration of 180 min and *APC* mode. The optimal predicted values are in good agreement with the experimental ones.

Based on these results, a central composite design can be developed in order to optimize the GAC-enhanced *EC* system. In our future work, we will consider *APC* mode of *EC* operation as an established improvement of this system. Only the continuous flow feature will allows us to estimate the reduction in the consumptions and costs of energy and electrode material in correlation with those of GAC material added.

### 3.5. Electrical Operating Costs

In order to determine these operational costs, economic data were gathered from the *EU* market in 2012. Thus the electrical energy price for industrial use is averaged at 0.1 $/kWh, while mild steel plate sheets were estimated at 1.5 $/kg [27].

As described in Section 2.3, Electrical Operational Costs (*EOC*s) are composed mainly of the costs of electrical energy and electrode material consumed. These costs were related to the amount of pollutant removed. The *EOC* can be determined by means of Equation (4).

According to the local optimum found by desirability function, treating an aqueous solution of 50 mg/L of dye at 2.73 A/m^2^, and adding 0.5 g/L of GAC dose and 26 mM of NaCl, a removal efficiency of 92.24% after 180 min was experimentally obtained. This corresponds to an *EOC* of 7.04 $/kg of pollutant removed. Under these conditions, it is important to note that the cost of energy is very low, namely about 0.018 $/kg of pollutant removed.

Future work should address the costs of adding GAC to *EC* systems. In order to achieve reliable data, this task must be performed for GAC-enhanced *EC* systems operated continuously as mentioned in the previous sections. Also, the possibility to integrate GAC-enhanced *EC* and electrochemical GAC regeneration could also be taken into account.

## 4. Conclusions

A *FFD* study was performed in order to achieve a better understanding of the effects of seven different parameters and their interactions on the performance of the *EC*/GAC coupling process. The contributions of alternating pulse current to the performance of an *EC* system enhanced by coupling with GAC adsorption were determined.

Current density, time and initial dye concentration factors show the most significant impact on the color removal efficiency, *UED* and *UEMD* respectively. Specifically, in the case of *UED* response, electrolyte support concentration also has a strong effect.

*APC* mode positively affects color removal efficiency response under certain experimental conditions, especially when the treated effluent has proper conductivity that favors the electrophoretic transport. Also, the addition of a GAC dose into an electrocoagulation reactor leads to enhancement of pollutant removal.

Based on the suggested models, local optimum values of the responses and their corresponding experimental conditions were established by means of a multi-objective desirability function method. Logical directions for future research include designing reliable GAC-enhanced *EC* reactors and studying their optimal operating parameters. Another important issue is to devise technical solutions for integration of GAC-enhanced *EC* and electrochemical GAC regeneration technologies.
